# An Update on New Generation Transcatheter Aortic Valves and Delivery Systems

**DOI:** 10.3390/jcm11030499

**Published:** 2022-01-19

**Authors:** Gloria Santangelo, Alfonso Ielasi, Mariano Pellicano, Azeem Latib, Maurizio Tespili, Francesco Donatelli

**Affiliations:** 1 San Paolo Hospital, Division of Cardiology, Department of Health Sciences, University of Milan, Via Antonio di Rudinì 8, 20142 Milan, Italy; gloriasantangelo@hotmail.it; 2 Clinical and Interventional Cardiology Unit, Istituto Clinico Sant’Ambrogio, Via Faravelli 16, 20149 Milan, Italy; marianopellicano@libero.it (M.P.); tespili@katamail.com (M.T.); 3 Division of Cardiology, Department of Medicine, Montefiore Medical Center, Albert Einstein College of Medicine, 111 210th Street, Bronx, NY 10467, USA; alatib@gmail.com; 4 Department of Cardiothoracic Center, Istituto Clinico Sant’Ambrogio, University of Milan, Via Faravelli 16, 20149 Milan, Italy; francesco.donatelli@unimi.it

**Keywords:** aortic stenosis, transcatheter aortic valve implantation, prosthetic aortic valves, aortic valve replacement

## Abstract

Over the last 15 years, the management of aortic valve disease has been changed by transcatheter aortic valve replacement, which has become the standard of care across the entire spectrum of surgical risk. As a result of continuous evolution of this technique, several next-generation transcatheter heart valves (THVs) have been developed to minimize procedural complications and improve patient outcomes. This review aims to provide an update on the new generation THVs and delivery systems.

## 1. Introduction

Transcatheter aortic valve replacement (TAVR), initially suited for inoperable patients with severe aortic valve stenosis [1] (AS), has become the standard therapy for patients older than 70 years irrespective of the surgical risk [2]. Due to the continuous technological evolution, several new generation transcatheter heart valves (THVs) have been designed by specifically incorporating features (i.e., lower profile, easier positioning/repositioning, and recapturability) to mitigate procedural complications such as paravalvular leak (PVL), vascular injuries, conduction disturbances, and to improve clinical outcomes [3,4,5]. Furthermore, the use of novel procedural tools (e.g., embolic protection devices, and vascular closure systems/techniques) are contributing to improving the outcome of TAVR. In this article, we provide an update on new generation THVs and delivery systems.

## 2. Transcatheter Heart Valves

The first human TAVR was successfully performed via a trans-septal approach in 2002 in patients with severe AS, multiple comorbidities, and cardiogenic shock [1]. The patient survived four months after the procedure before dying of unrelated complications. Trans-septal TAVR was feasible in almost 85% of (compassionate only) cases. In 2004, due to the acquisition of *Percutaneous Valve Technologies* by *Edwards Lifesciences*, TAVR reached an increasingly important clinical and commercial role.

The two predominant technologies for TAVR are balloon expandable (BE) THV and self-expanding (SE) THV systems. SE bioprostheses provide larger effective orifice area (EOA) and lower gradient, particularly in valves with a supra-annular design [6]. However, SE bioprostheses favor a higher percentage of new pacemaker implantation (PPM) and PVL than BE valves [7]. Clinical experience with TAVR is mostly related to the Edwards Sapien (*Edwards Lifesciences, CA, USA*) and Medtronic CoreValve (*Medtronic, CA, USA*) systems, but several newer THVs are competing in expanding markets. Improved THV sizing and design as well as smaller delivery systems have strongly reduced PVL and complications such as vascular injuries and bleeding [8]. As a consequence of the clinical experience gained over the years, the THV implantation procedure through the transfemoral route is now feasible in more than 90% of patients using low-profile sheaths and/or less traumatic, small-bore (approximately 14French) delivery systems [3]. Thus, the size of the access sheath required has significantly reduced from thee 22–24 French (Fr) of the first generation THVs to 14Fr of the new generation devices, reducing peri-procedural vascular complications rate. THV features have been progressively iterated over three generations of the BE Sapien and SE CoreValve Evolut to reduce PVL as well as improve valve hemodynamics. In the latest design of the Sapien 3, the Ultra, the height of the external polyethylene terephthalate skirt was increased by 40% with the aim to reduce PVL. 

### 2.1. Self-Expanding Transcatheter Heart Valves

The second generation **Evolut R** (ER) SE THV entered the market in 2014, providing a lower rate of PVL and higher device success compared to the CoreValve. ER included a 14Fr/16Fr delivery system for a sheathless approach while an 18Fr sheath is needed in the case of 23 to 29 mm ER implantation and 20Fr in case of a 34 mm ER implantation. A nitinol delivery catheter capsule that allows re-sheathing and recapturing of the THV during deployment, a novel nitinol design at the annulus that optimizes radial force, and a taller porcine pericardial sealing skirt are the main features of this bioprosthesis [9]. The latest iteration of the ER is the **Evolut Pro** (E-Pro). Its main highlight is an external pericardial wrap designed to reduce PVL while maintaining the features of the previous generation, self-expansion, and the ability to recapture and reposition the bioprosthesis [10]. Compared with the prior THV generation, a larger introducer sheath (16Fr) is needed (to check the potential transit of the delivery system using a sheathless approach) and a minimum vessel diameter of 5.5 mm (vs. 5 mm with ER) is needed. The **Evolut Pro+** has now resolved this issue. Evolut Pro+ is approved in four sizes: the 23-, 26-, and 29-mm can pass through arteries down to 5 mm, while the 34 mm system can pass down to 6 mm. The Evolut Pro+ has an outer porcine pericardial tissue wrap that increases surface area contact and tissue interaction between the THV and the native aortic annulus. It includes an inline sheath, allowing physicians to treat patients with a wide range of anatomies thanks to a favorable delivery profile. The SE E-PRO system has shown good outcomes in the prospective Medtronic Evolut PRO US Clinical Study and has been introduced into everyday practice [11]. Medtronic Evolut PRO US was a single-arm prospective U.S. registry enrolling 60 patients that were followed for 30 days. The primary endpoint was none or trace of paravalvular leak (PVL). In this study, the E-Pro system provided 98.3% in-hospital survival rates and excellent hemodynamics. Absence or trace PVL was reported in 72.4% of patients while the remaining 27.6% had mild PVL. No patient had moderate or severe PVL post-procedure and at 3-year follow-up [1,12]. Furthermore, a matched comparison of the E-Pro and ER devices [13] showed that both THVs are associated with favorable hemodynamic performance without significant differences in terms of mild PVL rate (<15% in both arms) and clinical outcome. In the FORWARD multicenter registry, mild PVL after TAVR using ER was shown in approximately 31% [14] of the cases whereas in the Evolut PRO registry [11], mild PVL was reported in almost 28% of subjects. Even the PPM rate has been shown to be lower with E-Pro compared with ER [11,15]. No significant differences in terms of other complications (according to the Valve Academic Research Consortium, VARC-2 criteria) were reported in any study comparing the two THVs.

The **Portico** (*Abbott Structural Heart, St Paul, MN, USA*) is a SE, fully re-sheathable and partially retrievable THV. It consists of three intra-annular, bovine pericardial tissue valve leaflets mounted in a radiopaque, SE nitinol frame with a sub-annular porcine pericardial sealing cuff to minimize PVL. Available valve sizes include 23, 25, 27, and 29 mm, allowing treatment of aortic valves with an annulus perimeter ranging from 60 to 85 mm. The delivery system is flexible, facilitating the transit through angulated aortic arch. The Portico THV features large stent cells to allow access to the coronary ostia. The inflow side of the stent frame is cylindrical with low extension into the LVOT, which may reduce conduction system trauma, lowering the risk for a PPM. In the PORTICO IDE trial [16], TAVR with Portico THV resulted was noninferior (for safety and efficacy end-points) compared with other THVs (e.g., BE Sapien, SE Evolut) among severe AS patients with extreme or high surgical risk. Portico was associated with higher PPM, moderate to severe PVL, and major vascular complication rates, while trans-prosthetic gradients were lower. A propensity-matched analysis including 177 patients treated by Portico vs. Sapien 3 [17] showed no statistically significant differences in terms of PPM (21.9% Portico vs. 17.5% Sapien 3, *p* > 0.05). However, a RCT involving 732 patients undergoing TAVR reported a higher rate of PPM with Portico compared with a pool, predominantly BE, of commercially available THV (27.7% Portico vs. 11.6% other THVs group) [18].

The SE **Navitor** THV is the iteration of Portico. The key innovation of this THV is an active outer fabric cuff designed to reduce the PVL. The cuff actively expands according to the phase of the cardiac cycle in order to fill the calcification related gaps between the virtual basal ring and prosthesis. The Navitor is implanted using Abbott’s *FlexNav delivery system* featured to improve the delivery, even in patients with minimal peripheral artery diameters (>5 mm). The 14Fr profile of the delivery system may be associated with a lower risk of vascular injuries. The performance of this new THV was evaluated in the Portico NG prospective, multi-center study (NCT04011722) in 120 patients with high or extreme surgical risk. Navitor implantation was associated with 0% 30-day mortality and absence or trace PVL in 80% of patients, with remaining 20% showing only mild PVL (any moderate-to-severe PVL was reported). Moreover, EOA was comparable to supra-annular THVs, and low trans-valvular gradients were reported. PPM rate was 15%, however, it should be underlined that the majority of patients presented with conduction disturbances at the baseline. The long-term (5-year) follow-up of this registry is ongoing while data on patients at low-to-intermediate risk treated by Navitor will be provided by the VANTAGE (NCT04788888) international, pre-market clinical trial.

**Hydra** (*Vascular Innovations Co., Ltd., Nonthaburi, Thailand*) was developed as a SE THV with a mechanism for recapturing during release. The Hydra consists of a SE nitinol stent frame with three leaflets positioned supra-annular of the native aortic valve and a sealing cuff (covering the proximal 12 mm of the inflow part of the frame) made of bovine pericardium. The three antennae on the outflow side of the stent frame allow for the fixation to the delivery system and after deployment, provide anchors at the outflow, which adapts to the aortic shape. The inflow, non-flared, part of the frame exerts a higher radial force than the outflow part to allow anchoring to the aortic annulus. The THV is available in three sizes: 22, 26, and 30 mm (annulus sizes between 18 and 28 mm). This SE THV can be fully recaptured, retrieved, and repositioned until 80 to 90% of deployment. The Hydra THV is implanted using the *Hydra Aortic valve delivery system*, which has a distal 18Fr capsule for the Hydra valve and a 12Fr shaft. The delivery system is introduced into an 18Fr sheath and is suitable only for retrograde implantation. Both the delivery and the loading system were the same for all three THV sizes. The GENESIS trial [19] assessed the performance of the SE Hydra THV system in patients with severe AS and high or extreme surgical risk. In this trial, VARC-2 defined device success was 92.5%. The immediate reduction in transvalvular gradient and improvement in EOA were confirmed at 6-month follow-up. Overall mortality rate at 30-day was 10%. The rates of PPM, major vascular injuries, and PVL were encouraging compared to contemporary SE THVs. There were no cases of THV embolization, stroke, or conversion to surgery. None of the patients had more than mild PVL at follow-up while the PPM rate was lower compared to a historical cohort of CoreValve/Evolut. This finding may be related to the lack of flaring at the inflow segment of the stent frame and a lower radial force of the THV compared to the counterparts. Furthermore, the wide cells in the stent frame favor coronary access, and at the same time, allow for a low profile delivery catheter, which may partly mitigate major vascular complications and strokes. More evidence on the Hydra THV performance came from the Hydra CE pre-market prospective study: 157 patients were enrolled in European and Asia-Pacific centers reporting a 30-day death rate of 7.0% (of whom 5.7% of cardiac causes), 30-day moderate/severe PVL rate of 6.3%, and 6.9% at 1-year while PPM rate was 11.7% at 30-days and 12.4% at 1-year [20].

The **Venus-A valve** (Venus Medtech) **and VitaFlow** (Microport) are novel THVs manufactured in China. Both have shown excellent performance in a complex cohort of patients with bicuspid aortic valve stenosis (BAV) [21]. The Venus A-valve is a SE THV comprising of a nitinol stent frame with supra-annular porcine pericardial leaflets while having a skirt to minimize the risk of PVL. Four sizes are actually available (23, 26, 29, and 32 mm). Wang et al. [22] demonstrated that the Venus A-valve has a higher rate of moderate/severe PVL than the E-Pro THV. However, after the completion of a learning phase, the results compared similarly with the commercially available SE THVs. These findings should be confirmed in randomized comparisons versus the other best-in-class THVs.

**VitaFlow** (MicroPort, Shangai, China) is the first approved THV to be made of bovine pericardial leaflets in China. The leaflets are pre-treated with the Vital-X™ technology that extracts phospholipids and cholesterol stabilizing collagen formation. This feature prevents valve leaflets from calcification, minimizes structural valve deterioration, and prolongs valve durability. It applies an innovative design of double layer skirts to more effectively reduce the occurrence of PVL. The 12-month clinical results following the treatment of 110 patients with severe AS including BAV demonstrate the safety and efficacy of this THV: overall death was 2.7%, major stroke 2.7%, major vascular complication 2.7%, coronary artery obstruction 1.8%, and PPM 19.1%. No moderate or severe PVL was reported [23]. **The VitaFlow Liberty™** is the next-generation iteration of the *VitaFlow^®^ Transcatheter Aortic Valve and Delivery System*. Inheriting the design from VitaFlow, VitaFlow Liberty features a hybrid density stent with double-layer skirts and a bovine pericardial leaflet. The retrievable delivery system of VitaFlow Liberty comes with a motorized handle, allowing for fast, stable, and accurate release and retrieval. In addition, VitaFlow Liberty is currently the only delivery system in the Chinese market whose distal end can be bent 360 degrees, providing superior flexibility to help minimize blood vessel damage and reduce the risk of complications. The motorized handle also provides operators with better control over the retrieval and repositioning of the valve.

#### 2.1.1. ACURATE neo™

The ACURATE neo™ (NEO) (Boston Scientific, Marlborough, MA, USA) system has been available for transfemoral TAVR in Europe since 2014. The **NEO** bioprosthesis is composed of porcine pericardial leaflets positioned on a SE nitinol stent frame in a supra-annular position, which favors large EOAs and low trans-valvular gradients [16,17]. The low radial strength and the minimal protrusion in the LVOT of the NEO frames reduce the possibility of damage to the conduction system [24,25]. This THV is deployed in a two-step, top-down release mechanism [26]. This unique implantation process reduces intra-procedural outflow obstruction (leading a theoretically higher hemodynamic stability during deployment) and allows for a stable implant without mandatory pacing. Three stabilization arches and the upper crown enhance co-axial alignment during deployment and THV stabilization. Furthermore, the upper crown may deter the native leaflets from the coronary ostia and reduce the risk of coronary occlusion [24,27]. This THV is actually available in 3 sizes (S 23 mm, M 25 mm, and L 27 mm for aortic annular diameters from 21 to 27 mm). The delivery system is compatible with a 14Fr sheath (iSleeve). Clinical studies have investigated the outcomes up to 12-months following NEO THV implantation for the treatment of severe tricuspid AS [25,28,29]. Malpositioning occurred in 0.5 to 2.2%, while the rate of coronary occlusion was negligible [30,31]. Based on the lower radial force of the nitinol frame of the NEO compared to other SE THVs, pre- and post-dilatation [26] are highly needed in cases with moderate-to-severe leaflet calcification. However, the lower radial strength may lead to less mechanical injury to the conduction system and explain the lower rate for a PPM implantation (from 8.3 to 12.3%9) [27,28,29] compared to the reported PPM following the implantation of other SE THVs (17.4% to 34.1%) [32,33]. On the other hand, the low radial force may translate into incomplete apposition of the device to the virtual basal ring, increasing the risk of PVL [19]. However, data from observational site-reported studies showed an incidence of moderate-to-severe PVL ranging from 1.4% to 4.8% [33,34,35]. Finally, 30-day overall mortality rate was reported as 0.6% to 3.4%, which was similar to the results following TAVR with other THVs [30,31,32,33,34,35]. The performance of the NEO in bicuspid AS was evaluated in a multicenter registry (n = 54 bicuspid vs. n = 658 tricuspid) [30]. Coronary occlusion was reported in 1.8% of the cases in the bicuspid group (1.8%). After propensity score matching, the trans-prosthetic gradient (9.8 ± 4.2 mmHg vs. 9.9 ± 4.5 mmHg, *p* = 0.9) and moderate-to-severe PVL rates (7.4% vs. 5.5%, *p* = 0.7) were similar between the two groups. However, PVL occurrence was significantly higher in the bicuspid cohort compared to an unmatched tricuspid group of subjects (7.4% vs. 3.1%, *p* = 0.0001), suggesting an increased risk of PVL in patients with BAV. An international study (n = 85 patients) using the NEO THV for the treatment of degenerated surgical aortic bioprostheses demonstrated that a “high” implantation of the NEO with the upper crown just above the stent posts of the degenerated bioprosthesis resulted in lower mean transvalvular gradients, but a higher rate of incomplete apposition (2.4%) and early valve degeneration [35]. The performance of the NEO THV was also evaluated in selected high-risk patients with pure AR. This pathology is often characterized by the absence of annular and leaflet calcification, making THV anchoring more difficult. Furthermore, the increased stroke volume secondary to AR and the presence of aortic root dilation may further increase the risk of THV migration [36]. In this context and in the absence of calcification, the protruding upper crown of NEO could function as a safety anchor to prevent its dislocation into the LVOT [31]. The SCOPE I was a randomized non-inferiority trial comparing NEO versus SAPIEN 3 in 739 patients with severe AS, evaluating a primary composite safety and efficacy endpoint at 30-days. NEO failed to meet non-inferiority compared to SAPIEN 3 with respect to the composite of overall death, any stroke, major bleeding, major vascular complications, coronary artery obstruction, acute kidney injury (AKI, stage 2 or 3), rehospitalization for THV-related symptoms or congestive heart failure, THV-related dysfunction requiring repeat procedure, moderate or severe PVL, or significant trans-prosthetic gradient within 30 days of the procedure [26]. The “failure” was mainly driven by a higher rate of moderate/severe PVL in the NEO group (9% vs. 3%, *p* < 0.0001). However, the trans-prosthetic gradient was lower and the mean EOA larger in patients treated with NEO vs. SAPIEN 3. The SCOPE I trial also suggested an increased risk of AKI in the NEO group potentially related to a relatively high contrast volume injection due to a higher need for pre- and post-dilatation [26]. Indeed, pre- and post-dilatation rates were higher when the NEO THV was implanted compared to other THVs [36,37,38]. Furthermore, a larger contrast volume and longer procedural time associated with the use of this THV compared to the SAPIEN 3 was reported [39,40]. The SCOPE 2 trial [41] (*Safety and Efficacy Comparison of Two TAVI Systems in a Prospective Randomized Evaluation 2*) was designed to compare the clinical outcomes of the NEO versus the SE CoreValve Evolut for the treatment of patients with severe native AS. The primary endpoint, powered for noninferiority of the NEO THV, was overall death or stroke at 12-month. The secondary end-points, powered for superiority of the NEO THV, was new PPM at 30-days. NEO failed to meet noninferiority compared with the SE CoreValve Evolut in terms of overall death or stroke at 1-year (15.8% vs. 13.9%, *p* = 0.0549), even if it was associated with a lower new PPM rate (10.5% vs. 18%, *p* = 0.0027). Furthermore, the NEO was associated with a higher rate of moderate/severe PVL at 30-days (10% vs. 3%; *p* = 0.002) and cardiac death at 30-days (2.8% vs. 0.8%; *p* = 0.03) and 1-year (8.4% vs. 3.9%; *p* = 0.01).

#### 2.1.2. ACURATE NEO2™

ACURATE NEO2™ is an iteration of the NEO THV and was approved in Europe in 2020. While both the NEO and NEO2 THVs have a pericardial sealing skirt to mitigate PVL risk, the skirt on the NEO2 is 60% taller, extends to the upper crown of the THV stent, is able to guarantee a more synchronous adaptation to the native virtual basal ring during the different phase of the cardiac cycle (active PVseal^TM^ technology), reaching the waist of the stent. Furthermore, the flexible NEO delivery catheter has been upgraded with a new (less traumatic) tip design, and when coupled with the low-profile expandable introducer, (iSleeve) is able to accommodate a wide range of anatomies. The NEO2 also has a new radiopaque marker to enhance visualization and accuracy during THV deployment. NEO2 sizing as well as crimping, loading, and release mechanism (top-down) remain unchanged compared to NEO. The radial force exerted by the stent frame remains low (compared to the predecessor), reducing the risk of mechanical injuries during deployment [24]. The ongoing ACURATE IDE trial (NCT03735667) will test the NEO2 THV versus other THV in all risk categories in the U.S. clinical and echocardiographic outcomes in patients treated with NEO2 THV were investigated in the NEO AS study (NCT02909556). Procedural success was high (97.5%) while vascular injuries relatively low (3.3%) [39,40,41]. Finally, the VARC-2 composite safety endpoint rate at 30-days was lower (13.3%) compared to prior studies (15.8% in the MORENA study; 16.4% in the NEOPRO study) with the NEO. Core laboratory-adjudicated data exhibited significant early hemodynamic improvement, maintained at 1-year [42,43]. The overall rate of moderate PVL at 30-days was 3.0%, similar to that reported with the Sapien 3 (3.6% by Mauri et al.; 2.8% in SCOPE I) and the Evolut R/PRO (3.0% in SCOPE II) [26,37,41]. Recently, promising data on real-world outcomes with the NEO2 was presented from two European registries. The ITAL-NEO Registry, which evaluated 220 patients treated with the NEO2, reported a low rate of in-hospital non disabling stroke (1%) and excellent valve hemodynamics [44]. In this study, 3.5% of patients exhibited moderate PVL at discharge, 46.5% had mild PVL, and 50% had none/trace PVL. Any severe PVL was reported. Compared to NEO (n = 680 patients), NEO2 was associated with a more than 3-fold reduction in PVL (11.7% vs. 3.5%, *p* < 0.001). The Early NEO2 Registry (NCT04810195) enrolled 554 patients treated with the NEO2 in several European centers. In-hospital stroke (2.1%) and PPM rates (6%) were relatively low, while NEO2 hemodynamics was excellent (post-procedural mean gradient of 9 mmHg) and 30-day mortality rate low (1.3%) [45,46]. According to the post-procedure echocardiographic data, 1.3% of patients had moderate/severe PVL, 33.3% showed mild PVL, and 65.4% had no/trace PVL [38]. This study also included a retrospective analysis of core-lab adjudicated echocardiographic data from patients treated with NEO (n = 108) versus NEO2 (n = 120) [40]. NEO2 was associated with a 5.5% absolute risk reduction in PVL fraction (*p* < 0.001) and the rate of moderate or severe PVL was significantly lower with the NEO2 compared to NEO (1.7% vs. 13.9%; *p* < 0.001) [46].

### 2.2. Self-Expanding Transcatheter Heart Valves for Aortic Regurgitation

The **JenaValve (JenaValve Technology, Germany) THV** consists of a porcine root valve sewn onto a nitinol SE stent that has three feelers that are designed to embrace the native valve cusps during deployment [47]. This mechanism is expected to provide tactile feedback during THV positioning and allow for coaxial prosthesis implantation. After placing the feelers in the correct position in the sinuses of the native valve, the lower part of the THV is released. The nitinol stent self-expands to anchor in the virtual basal ring, and the THV immediately starts to function. During release, the native valve leaflets are clipped between the feelers and the base of the prosthesis. This mechanism firmly anchors the JenaValve in the correct anatomical position and provides active fixation and resistance to migration. This mechanism provides stability in the setting of pure AR. The JenaValve system is deployed trans-apically through a 32Fr sheath and is fully repositionable and retrievable. This THV is available in three sizes: 23 mm, 25 mm, and 27 mm (annuli from 21 mm to 27 mm). A multicenter CE-mark study on 73 patients showed a 30-day overall mortality rate of 7.6% (3% cardiac death), stroke rate of 3%, and a PPM implantation rate of 9.1% [48]. No cases of moderate-severe PVL were reported. Based on these results, this THV received the Conformite’ Europèene (CE) mark of approval for trans-apical implantation in September 2011. The *JenaValve transfemoral first-in-man* study was started in 2012 with the aim to assess the performance of this device through the transfemoral approach, but was stopped due to the low enrollment rate. Technical success was 93.3%. None/trace post-procedure PVL was 35.7% while mild was 64.3%. At 30-days, none/trace PVL was reported in 63% of the patients and mild in 37%. No moderate or severe PVL was reported. One patient required PPM while any death was reported at 30-days. In the JUPITER (*JenaValve EvalUation of Long Term Performance and Safety In PaTients with SEvere Aortic Stenosis oR Aortic Insufficiency*) study, the authors reported satisfactory outcomes using the JenaValve for predominant AR, as no annular rupture or coronary obstruction occurred. Mortality at 30-day was 10%, PVL was none/trivial in 84.6%, and mild in 15.4% while PPM implantation rate was 3.8%. Survival at 1-year was 79.9% while any stroke was observed at 1-year [49].

The **J-valve (JieCheng Medical, China**) is a second generation THV device consisting of porcine aortic leaflets attached to a SE nitinol stent and three U-shape anatomically oriented “graspers” encircling the stent. These features could facilitate intuitive “self-positioning” THV deployment and provide both axial as well as radial fixation by embracing the native leaflets. Based on the anchoring mechanism, the THV is partially retrievable and repositionable. Four sizes are available: 21, 23, 25, and 27 mm (aortic annuli from 19 to 27 mm). Zhu et al. showed that transapical TAVR using this THV is a treatment option for patients with AS or pure/dominant AR at high surgical risk [50].

### 2.3. Balloon-Expandable Transcatheter Heart Valves

The **Myval** THV (Meril Life Sciences, Gujarat, India) is a BE device that obtained the CE mark in 2019. This THV consists of a nickel–cobalt alloy (MP35N) frame, which enables high radial force and radiopacity, and bovine pericardium tissue, crafted into a tri-leaflet valve. This THV has a hybrid honey-comb scaffold design. The upper part of the frame is composed of a single row of tall, large, and open-cell configuration to ensure unjailing of the coronary ostia that preserves coronary flow; the lower part of the frame is composed of two short rows of tightly packed, close-cell hexagonal configuration, providing the high radial force required at the annular base. This design allows for precise deployment of the THV and guarantees its orthotopic implantation. The lower closed cells of Myval THV are covered externally with a polyethylene terephthalate sealing cuff made to form an external buffing zone that reduces the risk of PVL [51,52]. Myval is available in many sizes: conventional (20, 23, 26, and 29 mm), medium (21.5, 24.5, and 27.5 mm), and extra-large (30.5 and 32 mm). This THV is crimped over the balloon (*Mammooth*) outside the patient. All Myval sizes are compatible with 14Fr expandable sheaths (*Python*). The *Navigator* delivery system features a proximal deep flexion handle and a distal balloon with two counter opposing stoppers within that create a superficial low-profile crimping zone and thus a comfortable fit that prevents any unwary dislodgement of the Myval THV during crossover through the sheath or later. The delivery system allows for flexion of the distal catheter system that ensures trauma-free negotiation across the aortic arch and mitigates the risk of a peri-procedural stroke during arch navigation [53]. The first-in-human MyVal-1 study demonstrated the safety and efficacy of this BE implanted using a percutaneous trans-femoral approach in 30 patients with a low incidence of 30-day as well as 12-month all-cause mortality (four out of 30 patients) [51]. No new PPM was required during or after the implantation of the Myval THV. Additionally, no moderate/severe PVL, thrombosis, or THV migration was reported at 1-year follow-up. Data coming from a quantitative videodensitometric angiographic aortic regurgitation assessment following TAVR with Myval (n = 108 patients) versus Sapien 3 (n = 397 patients) and Sapien XT (n = 239 patients) THVs demonstrated a numerically lower occurrence of paravalvular AR vs. Sapien 3 (6.3% ± 6.3% vs. 7.6% ± 7.1%, *p* = 0.2) while a significantly lower rate versus Sapien XT (8.8% vs. 7.5%, *p* = 0.006) was reported [54].

Delgado-Arana et al. [55] compared Sapien 3 THV (chromium-cobalt frame) vs. Myval THV (nickel-cobalt frame) in 416 severe AS patients with the aim to assess the 30-day clinical outcomes and hemodynamic performance through central blinded imaging analysis after matching for main baseline and anatomical characteristics. The authors found that the use of the Myval was associated with favorable early safety (12.6% vs. 4.9%, *p* = 0.09) and clinical efficacy (12.6% vs. 4.9%, *p* = 0.05) when compared with the Sapien 3 at 30-day follow-up. Furthermore, lower trans-valvular gradients (12 mmHg Sapien-3 vs. 8 mmHg Myval, *p* < 0.001) and similar PVL (1% Sapien-3 vs. 0% Myval, *p* = 0.314) were reported with the Myval, suggesting a device-related effect and indicating the potential benefits of the intermediate sizes (chosen in 44.6% of the patients). Finally, the rate of PPM was significant with the Myval THV. In this view, recent registry data (n = 1131 patients) showed that Myval THV was associated with a lower rate of early conduction disturbances (blinded ECG analysis) compared to six commercially available THVs [56]. Hemodynamics and conduction disturbance rate are part of the primary composite endpoint of the ongoing randomized controlled LANDMARK trial [57] (NCT04275726) that will recruit 768 patients worldwide allocated in a 1:1 randomization to Myval, Sapien, or Evolut. Long-term clinical follow-up (10 years) will provide information on the long-term durability of this novel BE THV compared to the SAPIEN or Evolut series.

### 2.4. Investigational Devices

The **Colibri** THV (Colibri Heart Valve LLC, Louisville, CO, USA) shows conical rather than spherical cusp geometry, with an elliptical leaflet cross-section that progressively decreases moving proximal to the plane of leaflet apposition while being readily conformed on outward radial compression in the THV opening phase into a substantially flat folded construct against the interior tubular walls of the containing frame. These characteristics allow large EOA and low trans-valvular energy losses. Furthermore, the conical cusps are particularly suited for compression and containment within a collapsible frame for transcatheter delivery. Each cusp and leaflet structure are attached to the frame and simultaneously closed along a seam that is non-load-bearing. The sutures are principally directed to mounting of the cusps and therefore number far fewer than in existing THV designs, with current production models utilizing only approximately 150 sutures. In addition, the reduction in the number of sutures reduces the material mass within the frame envelope, favoring the compression and low profile of the mounted device. In order to achieve a dry, thin crimped tissue that confers a lower mass and profile, high tensile strength is required. Crimping and mounting in the dry state impose high stresses upon the tissue material, which require that the tissue material should possess equally high tolerance to these stresses. Typical valve leaflet tissue materials have ultimate tensile strength, or breaking stress values in the 6–8 megaPascal (mPa) range with 12 mPa representing a very high stress performance. In response to this design requirement, Colibri has developed tissue materials with ultimate tensile strengths in the 30–50 mPa range. It should be emphasized that this performance is not required for the operation of the valve in vivo (requiring only 10s of kiloPascals), but rather for the tolerance of forces imposed by the crimping and mounting in the dehydrated state. Following the general design principles as outlined above, the Colibri THV was created as a successful embodiment of a dry, pre-mounted, pre-packaged THV that is encapsulated upon a delivery balloon catheter within a 14Fr introducer sheath and packaged integrated upon the delivery catheter ready to use. In the first human case [58] of transfemoral implantation of the Colibri pre-mounted and prepackaged THV in its 14Fr delivery sheath, clinical outcome at 6-months after implantation showed sustained benefits.

**Figure 1:**
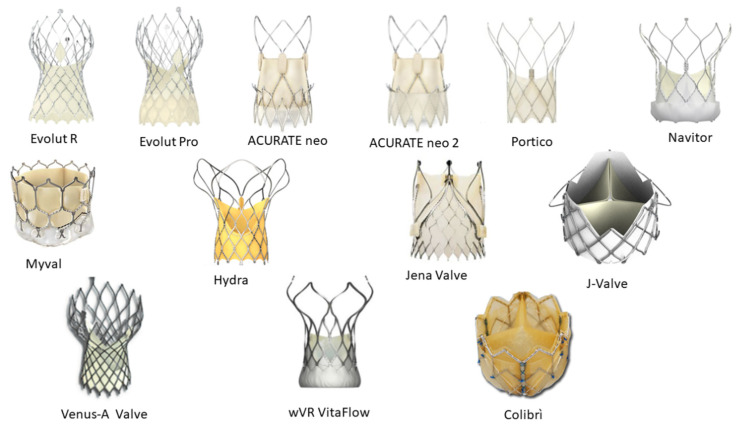
New generation THVs.

**Figure 2:**
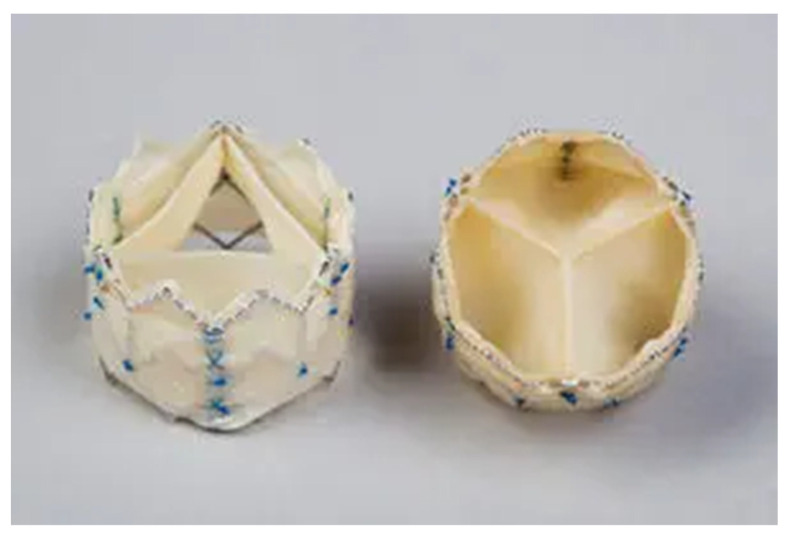
The Colibri valve is a dry-porcine, pre-mounted, pre-packaged BE THV, encapsulated upon a delivery balloon catheter within a 14Fr introducer sheath and packaged integrated upon the delivery catheter ready to use.

In Table 1, we summarize the characteristics of the available THV devices. In Figure 1, we show the THV types mentioned in the text. In Figure 2, we present the characteristics of the Colibri THV.

## 3. Conclusions

TAVR has transformed the management of AS over the years, establishing itself as the standard therapy for patients with symptomatic severe AS older than 70 years, irrespective of the surgical risk. Continuous technological evolution and the use of new procedural additions continuously optimize the predictability, safety, and outcomes of the procedure.

## Figures and Tables

**Table 1:** Characteristics of the available transcatheter heart valve devices.

THVs	Company	Access Route	Sizes (mm)	Stent Frame	Delivery System Diameter (Fr)	Leaflets Tissue	Repositionable	Retrievable	PPM (%)	PVL (%)	Major Vascular Complications (%)
Evolut Pro	Medtronic	TAo, TV	23, 26, 29	Nitinol	16	Porcine	Yes	Yes	10.8 *–11.9 *	0 *–11 *	0–10
NEO2	Boston Scientific	TA, TV	23, 25, 27	Nitinol	14	Porcine	Yes	No	6.0 *–7.7 *	2.7 *–3.5 *	3.4
Portico	Abbott	Tao, TV	23, 25, 27, 29	Nitinol	18	Bovine	Yes	Yes	8.8 *–15.8 *	3.4 *–5.7 *	2.2–7.2
Navitor	Abbott		23, 25, 27, 29	Nitinol	14	Bovine	Yes	Yes	15 *	0 *	0.8
Myval	Meril Life Sciences	TV	20, 23, 26, 29, 21.5, 24.5, 27.5,30.5, 32	Ni-Co	14	Bovine	No	No	5.8 *–8 *	0 *–4 *	0–3
Hydra	Vascular Innovations	TV	22, 26, 30	Nitinol	18	Bovine	No	No	7.5 *	10 *	2.5
JenaValve	Jena Valve	TA	23, 25, 27	Nitinol	32	Porcine	Yes	Yes	9.1 *		0
J-valve	Jie Cheng Medical Technologies	TA	21, 23, 25, 27	Nitinol	27	Porcine	Yes	Yes	2.3 *–4.8 **	0	0
Venus-A valve	Venus Medtech	TV	23, 26, 29, 32	Nitinol	19	Porcine	Yes	No	7.4 *–18.8	8.8 *–14.2 *	6.2
wVR VitaFlo	Microport	TV	21, 24, 27, 30	Nitinol	16/18	Bovine	Yes	No	16.4 *–19.1 **	2 *	1.8
Colibri	Colibri Heart Valve	TV	24	Stainless Steel	14	“Dry” porcine	No	No	NA	NA	NA

AR: Aortic regurgitation. Ni-Co: Nickel–Cobalt. NA: Not available. PPM: permanent pacemaker. PVL: equal or more than moderate paravalvular regurgitation. THV: Transcatheter heart valves. Fr: French. TA: transapical. TAo: transaortic. TV: transvascular. * 30-day results; ** 1-year results.

## Data Availability

Data sharing not applicable. No new data were created or analyzed in this study. Data sharing is not applicable to this article.
